# A cross-sectional study evaluating hospitalization rates for chronic limb-threatening ischemia during the COVID-19 outbreak in Campania, Italy

**DOI:** 10.1177/1358863X20977678

**Published:** 2020-12-17

**Authors:** Eugenio Stabile, Raffaele Piccolo, Michele Franzese, Giancarlo Accarino, Umberto Marcello Bracale, Enrico Cappello, Giovanni Cioffi, Angelo Cioppa, Adolfo Crinisio, Loris Flora, Pietro Landino, Eugenio Martelli, Rosario Mancusi, Raffaella Niola, Fernando Petrosino, Davide Razzano, Carlo Ruotolo, Luigi Salemme, Paolo Sangiuolo, Gianpaolo Santini, Emilio Soreca, Gennaro Vigliotti, Bruno Villari, Giampaolo Amabile, Raffaele Pio Ammollo, Danilo Barbarisi, Alfonsina M Corbisiero, Antonio D’angelo, Gianluca Cangiano, Claudia De Gregorio, Mario De Laurentis, Eugenio Laurenzano, Ilaria Ficarelli, Alessandro Luongo, Claudio Molino, Giuseppe Sarti, Daniela Viola, Giovanni Esposito

**Affiliations:** 1 Department of Advanced Biomedical Sciences, Division of Cardiology, University of Naples Federico II, Naples, Italy; 2 Department of Vascular and Endovascular Surgery, AOU San Giovanni di Dio e Ruggi d’Aragona, Salerno, Italy; 3 Department of Public Health, Division of Vascular Surgery, University of Naples Federico II, Naples, Italy; 4 Division of Vascular and Endovascular Surgery, Mediterranea Centro Cuore, Naples, Italy; 5 Division of Vascular Surgery, Ospedale Pellegrini, Naples, Italy; 6 Division of Invasive Cardiology, Clinica Montevergine, Mercogliano, Italy; 7 Division of Vascular and Endovascular Surgery, Clinica Salus, Battipaglia, Italy; 8 Division of Vascular Surgery, AORN San Giuseppe Moscati, Avellino, Italy; 9 Department of Cardiology and Cardiac Surgery, Casa di Cura S. Michele, Maddaloni, Italy; 10 Division of Vascular and Endovascular Surgery, AORN Sant’Anna e San Sebastiano, Caserta, Italy; 11 Division of Vascular and Endovascular Surgery, Villa dei Fiori Hospital, Acerra, Italy; 12 Division of Vascular and Interventional Radiology, AORN Cardarelli, Naples, Italy; 13 Division of Vascular Surgery, Presidio Ospedaliero ‘San Luca’, Vallo della Lucania, Italy; 14 Division of Vascular Surgery, AO San Pio, Benevento, Italy; 15 Division of Vascular Surgery, AORN Cardarelli, Naples, Italy; 16 Division of Vascular Surgery, AORN Ospedali dei Colli, Monaldi Hospital, Naples, Italy; 17 Department of Radiology, Ospedale del Mare, Naples, Italy; 18 Department of Radiology, AO San Pio, Benevento, Italy; 19 Division of Vascular Surgery, Ospedale del Mare, Naples, Italy; 20 Division of Cardiology, Ospedale Sacro Cuore di Gesù, Benevento, Italy

**Keywords:** chronic limb-threatening ischemia (CLTI), COVID-19, peripheral artery disease (PAD)

## Abstract

The expansion of coronavirus disease 2019 (COVID-19) prompted measures of disease containment by the Italian government with a national lockdown on March 9, 2020. The purpose of this study is to evaluate the rate of hospitalization and mode of in-hospital treatment of patients with chronic limb-threatening ischemia (CLTI) before and during lockdown in the Campania region of Italy. The study population includes all patients with CLTI hospitalized in Campania over a 10-week period: 5 weeks before and 5 weeks during lockdown (*n* = 453). Patients were treated medically and/or underwent urgent revascularization and/or major amputation of the lower extremities. Mean age was 69.2 ± 10.6 years and 27.6% of the patients were women. During hospitalization, 21.9% of patients were treated medically, 78.1% underwent revascularization, and 17.4% required amputations. In the weeks during the lockdown, a reduced rate of hospitalization for CLTI was observed compared with the weeks before lockdown (25 vs 74/100,000 inhabitants/year; incidence rate ratio: 0.34, 95% CI 0.32–0.37). This effect persisted to the end of the study period. An increased amputation rate in the weeks during lockdown was observed (29.3% vs 13.4%; *p* < 0.001). This study reports a reduced rate of CLTI-related hospitalization and an increased in-hospital amputation rate during lockdown in Campania. Ensuring appropriate treatment for patients with CLTI should be prioritized, even during disease containment measures due to the COVID-19 pandemic or other similar conditions.

## Introduction

The ongoing coronavirus disease 2019 (COVID-19) outbreak is poised to challenge populations and healthcare systems the world over. A reduction in hospital admissions for cardiovascular disease has been observed globally as a consequence of the pneumonia outbreak caused by COVID-19.^1^ Despite the emergence of reports on the management of cardiac disease, few data have been reported so far on the management of peripheral artery disease (PAD) during the COVID-19 outbreak.^2,3^

Among PAD-related syndromes, chronic limb-threatening ischemia (CLTI) is more frequently associated with the need for urgent treatment and a delay in providing care could result in adverse cardiovascular and limb-related events.^4^

The sheer speed of geographical expansion of COVID-19 throughout Italy, coupled with the high number of cases requiring hospitalization or admission to the intensive care unit, prompted important measures of disease containment by the Italian government – resulting in lockdown of the entire country by March 9, 2020.

As a possible consequence of the lockdown, patients with acute CLTI tended not to report or were dismissive of their symptoms, resulting in a slower or even no activation at all of the emergency system. Similarly, patients with chronic conditions may have encountered difficulties in receiving the proper care by the usual network of physicians because of the limitations imposed by the measures for disease containment.^5^

The aim of the study is to evaluate the trends in hospitalization for CLTI as well as its management before and during the COVID-19 outbreak in Campania in order to evaluate whether containment measures have temporally impacted on the treatment of this disabling and life-threatening condition.^6^

## Methods

### Study design and population

This cross-sectional study obtained data from the 20 centers that routinely hospitalize patients with CLTI in the Campania region. These hospitals represent all the facilities that treat patients with CLTI in Campania. The observation period lasted 10 weeks, and included the 5 weeks before (before lockdown period) and the 5 weeks after March 9, 2020 (during lockdown period), corresponding to the date of the lockdown in Italy. The present analysis included only patients who were hospitalized for CLTI in Campania. The protocol of the study was approved by the Ethics Committee of the University of Naples Federico II (Italy) and complies with the Declaration of Helsinki.

### Data collection management

This cross-sectional study used a registry that collects data on sex, age, disease status, type of treatment, and amputations. Patients’ related data were directly collected from each hospital. The presented data depicts the characteristics and outcomes of hospitalized patients (either with same-day discharge or with overnight hospitalization) for CLTI-related issues in Campania during the study period. All the patients hospitalized in the enrolling institutions signed an informed consent that allowed the collection and management of anonymized data.

CLTI is defined as ischemic rest pain, tissue loss, or gangrene in the presence of PAD and hypoperfusion.^4^ The Fontaine classification systems have been used to classify patients with CLTI. In particular, we considered Fontaine stage 3 for patients who required hospital admission because of ischemic rest pain and Fontaine stage 4 for patients who already had ulceration or gangrene at the time of hospitalization.^5^

Among the measures introduced by the Italian government during lockdown was the constraint of traveling from home only for urgent reasons related to work, health or supplies. Concerning the practice of hospital clinics, all non-urgent elective activity was suspended while urgent hospitalizations, through the emergency room or referral centers (i.e. critical limb ischemia clinics), were performed regularly.

According to clinical and anatomic evaluation, patients were treated medically or underwent an urgent revascularization. Revascularization was performed using an endovascular and/or surgical approach. Hybrid treatment was defined as the combination of endovascular and surgical revascularization in the same patient.

Amputation was defined as any procedure resulting in an amputation of the lower extremities.

### Outcome

The primary outcome was the rate of CLTI-related hospitalization during the COVID-19 pandemic. Secondary outcomes were revascularization rates and primary amputation rates.

### Statistics

Statistical analyses were performed using SPSS, version 26.0 (IBM Corp., Armonk, NY, USA). Normality of distributions were tested using the Shapiro–Wilk test. Nominal and categorical variables are presented as contingency tables with frequencies and percentages. Continuous variables are presented as mean with SD and were compared with the Wilcoxon rank sum test or *t*-test (probability value < 0.05 was considered statistically significant). Proportions were compared by chi-squared or Fisher exact tests. The rate of CLTI-related hospitalization and their ratios were calculated using Poisson regression analysis.^6^ Population denominators, which were used as offset, were obtained from the Italian census. A two-tailed probability value < 0.05 was considered statistically significant. A Cochran–Armitage test was used to assess linear trend over weeks.

## Results

A total of 453 patients were hospitalized for CLTI at 20 centers from February 3, 2020 to April 13, 2020. The mean age was 69.2 ± 10.6 years and 27.6% of the total patients were women.

Over the entire study period, at hospital admission, 39.1% (*n* = 177) of patients were at Fontaine stage 3 and 60.9% (*n* = 276) were at stage 4. During hospitalization, 21.9% (*n* = 99) were treated medically and 78.1% (*n* = 354) underwent an urgent revascularization. Amputation was necessary in 17.4% (*n* = 79) of patients (Table 1).

**Table 1.** Demographic and clinical characteristics of the study population.

	Overall	Lockdown		*p*-value
		Before	During	
	*n* = 453	*n* = 337	*n* = 116	
Age, mean ± SD, years	69.2 ± 10.6	68.9 ± 10.8	70.2 ± 10.1	0.242
**Age group, *n* (%)**				
< 55 years	40 (8.8)	32 (9.5)	8 (6.9)	0.453
55–64 years	99 (21.9)	77 (22.8)	22 (19.0)	0.436
65–75 years	181 (40.0)	133 (39.5)	48 (41.4)	0.742
> 75 years	133 (29.4)	95 (28.2)	38 (32.8)	0.348
Female, *n* (%)	125 (27.6)	89 (26.4)	36 (31.0)	0.338
Fontaine classification, *n* (%)				
Stage 3	177 (39.1)	145 (43.0)	32 (27.6)	0.004
Stage 4	276 (60.9)	192 (57.0)	84 (72.4)	0.004
In-hospital management, *n* (%)				
Medical therapy	57 (12.6)	43 (12.8)	14 (12.1)	1
Urgent revascularization	317 (70.0)	249 (73.9)	68 (58.6)	0.003
Urgent revascularization and amputation	37 (8.2)	22 (6.5)	15 (12.9)	0.047
Medical therapy and amputation	42 (9.3)	23 (6.8)	19 (16.4)	0.005
Revascularization, *n* (%)	354 (78.2)	271 (80.4)	83 (61.5)	0.001
Revascularization group, *n* (%)				
Percutaneous	275 (77.7)	217 (80.1)	58 (69.9)	0.07
Surgical	73 (20.6)	50 (18.5)	23 (27.7)	0.087
Hybrid	6 (1.7)	4 (1.5)	2 (2.4)	0.628
Amputation during hospital stay, *n* (%)	79 (17.4)	45 (13.4)	34 (29.3)	< 0.001

*Overall*: all patients included in the study; *Before*: patients hospitalized during the 5 weeks before regional lockdown; *During*: patients hospitalized during the 5 weeks during the national lockdown; *Hybrid*: endovascular + surgical.

**Figure 1.**
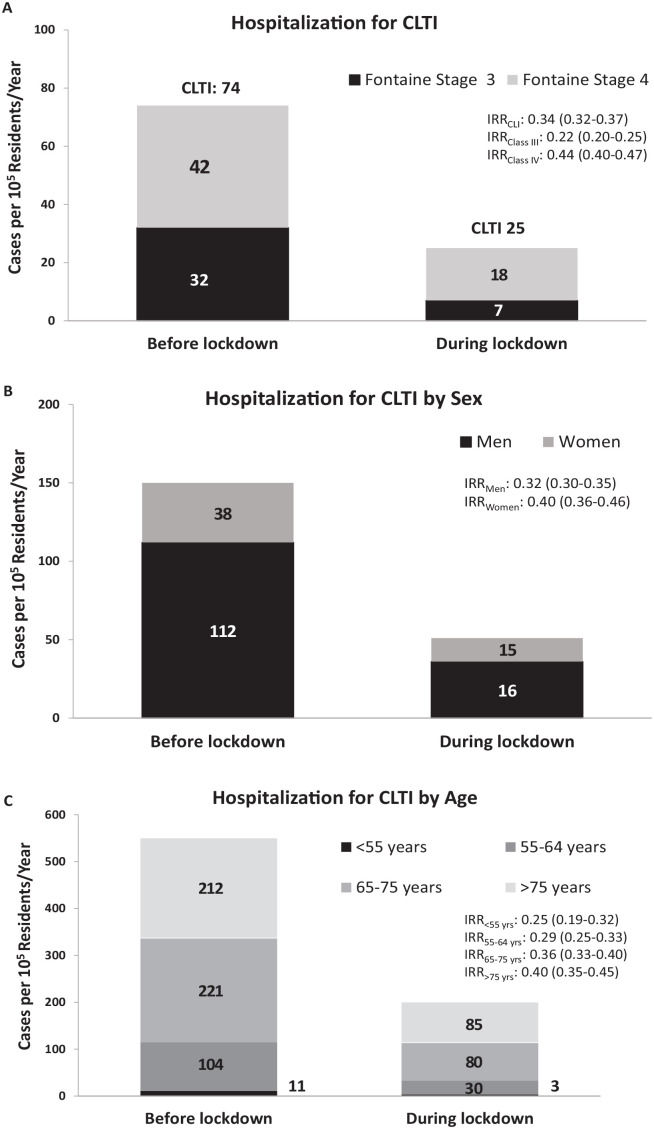
Representative bar graph indicating the rate of CLTI-related hospitalization before and during the lockdown in the Campania region (March 9, 2020) (A) according to Fontaine stage 3 or 4; (B) according to sex; and (C) according to age categories. CLTI, chronic limb-threatening ischemia; IRR, incidence rate ratio.

During the observation period, we observed a severe reduction in the rate of CLTI-related hospitalization. This moved from 74 cases/100,000 residents/year in the 5 weeks before the lockdown to 25 cases/100,000 residents/year in the 5 weeks during the lockdown. The variation of the rate of CLTI-related hospitalization is indicated by an incidence rate ratio (IRR) of 0.34 (95% CI 0.32–0.37) (Figure 1A).

A similar reduction was observed in men (IRR 0.32, 95% CI 0.30–0.35) and women (IRR 0.40, 95% CI 0.36–0.46) (Figure 1B).

The decrease in hospitalization rates for CLTI was consistent across age categories, although the decline in CLTI admission rates was slightly less pronounced with the aging of the population (Figure 1C).

The decrease in CLTI-related hospitalization was more evident among patients with a Fontaine stage 3 (IRR 0.22, 95% CI 0.20–0.25) than among those with a Fontaine stage 4 (IRR 0.44, 95% CI 0.40–0.47) classification (Figure 1A). In particular, the rate of patients who presented with Fontaine stage 4 was higher in the weeks during the lockdown and a reduced rate of urgent revascularization coupled with an increase in the hospital amputation rate was observed (29.3% vs 13.4%; *p* < 0.001) (Table 1). No repeated hospitalization was observed in the study population.

**Figure 2.**
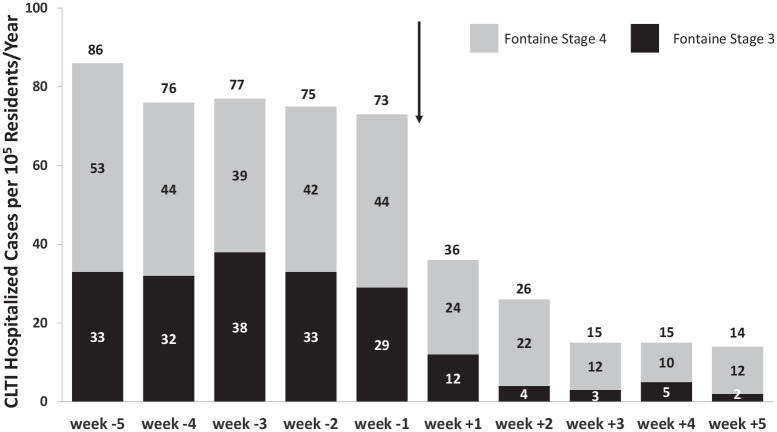
Representative bar graph indicating the rate of CLTI-related hospitalization by week according to Fontaine stage 3 or 4. Weeks −5 to −1 represent the 5-week period before the lockdown in the Campania region (up to March 9, 2020) and weeks 1 to 5 represent the 5-week period during the lockdown in the Campania region (from March 9, 2020) (arrow). The values indicate the incident rates by week according to the Fontaine stage 3 or 4. CLTI, chronic limb-threatening ischemia.

When the analysis was conducted on a weekly basis, we observed a trend to a progressive reduction in CLTI hospitalization throughout the lockdown period for all CLTI cases, as well as for single Fontaine stages (*p* for trend < 0.001) (Figure 2).

## Discussion

This study reported that in the 5 weeks during the lockdown of the Campania region of Italy:

- The rate of CLTI hospitalization was reduced to 1/3 of those observed in the 5 weeks before the lockdown.
- The amputation rate of patients hospitalized with CLTI was increased, when compared to that observed before the lockdown.

In the third most populous region of Italy, we found evidence that the lockdown during the COVID-19 pandemic was associated with a remarkable decline in the number of patients hospitalized for CLTI. Only a small portion of the patients treated were women. This can be related to the reduced incidence of PAD in women in Italy.^5^

Mechanisms underlying this decrease are unknown, although several explanations might be involved. For instance, ischemic rest pain might not be reported by patients with acute conditions due to fear of exposure to COVID-19-affected individuals at hospital admission, as has already been demonstrated in patients with acute coronary syndromes.^1,7,8^

In Campania, patients with CLTI are regularly treated in hospital within dedicated departments where they can be admitted through direct access to the emergency room or emergently transferred from an ambulatory setting or from another hospital that does not have dedicated divisions for the care of these patients. No variations in patients’ admitting pathways were observed in the study period.

Potential reasons for the decrease in CLTI hospitalizations include patients’ avoidance of medical care owing to social distancing or concerns over contracting COVID-19 in the ambulatory setting – a situation which can be more relevant for those patients with chronic conditions, such as the CLTI population, who often need wound care, podiatry and dialysis services, and diabetic care on an outpatient basis.^9^ Access to primary care and these ancillary services was not significantly impacted by the government lockdown-related changes to the Italian healthcare system, but patients were potentially reluctant to seek these services due to fear of COVID-19 infection in healthcare settings.

Our data reported that, in the weeks following the implementation of measures for disease containment and the national lockdown, there was a substantial reduction in the rates of revascularization, an increase in the proportion of patients hospitalized with more severe clinical conditions, and an increased amputation rate among patients with CLTI.

During the lockdown, patients were more frequently admitted with already established tissue loss or gangrene that could not benefit from revascularization procedures and more frequently underwent amputation. We cannot exclude that, during the lockdown period, some chronic conditions, such as diabetes, could have been treated less efficiently and resulted in poor metabolic control (i.e. thus precipitating the gangrene).^9^

A clinical factor that may have contributed to the observed data is the need to reduce in-hospital stay in order to reduce the possibility of nosocomial infection. During the COVID-19 pandemic period, this approach has not been limited to a single condition (i.e., patients with CLTI), but was a strategy that had to be adopted for the treatment of all severely ill cardiovascular patients.^10^

### Study limitations

This study is retrospectively observing a limited and selected patient cohort. There are no data about the patients that were not treated in hospital because our dataset includes only patients that were admitted in the study institutions. Although this study involved all the centers that routinely hospitalized patients with CLTI in the Campania region, we cannot exclude that some cases could have been hospitalized in other centers not routinely involved in the care of patients with CLTI. Although this possibility is quite rare – in Campania, if a patient with CLTI is admitted to a hospital that does not have a dedicated division, the case is routinely transferred to an institution with a dedicated division – we cannot completely exclude the issue of residual confounding cases.

It could have been important to obtain objective vascular measures (i.e., ankle–brachial index) in both time periods, particularly since classification schemes such as Fontaine can be subjective and not clearly differentiate degrees of tissue loss. This may help to determine if the increase in amputations was due to alterations in physician practice versus true change in disease severity at presentation.

## Conclusions

This study reports a reduced rate of CLTI-related hospitalization and an increased in-hospital amputation rate during lockdown in Campania. The findings of this study might have important implications for healthcare systems and suggest that public campaigns aiming to increase awareness of CLTI-related morbidity/mortality should be reinforced, even during the COVID-19 pandemic or other possible future similar conditions.^11^
